# Validity and reliability of the Australian Therapy Outcome Measures – Physiotherapy, for podiatry (AusTOMs-PT for use in podiatry)

**DOI:** 10.1186/s13047-020-00385-0

**Published:** 2020-04-25

**Authors:** Cylie M. Williams, Nina Davies, Jessica Kolic, Antoni Caserta, Alicia M. James, Carolyn Unsworth

**Affiliations:** 1 Allied Health, Peninsula Health, 4 Hastings Rd, Frankston, VIC 3199 Australia; 2 Department of Physiotherapy, Monash University, McMahons Rd, Frankston, VIC 3199 Australia; 3 Faculty of Health and Sciences, Staffordshire University, Leek Road, Stoke-on-Trent, ST4 2DF UK; 4 Podiatry Department, Peninsula Health, 4 Hastings Rd, Frankston, VIC 3199 Australia; 5 School of Health, Medical and Applied Sciences, Central Queensland University, 120 Spencer St., Melbourne, VIC 3000 Australia

**Keywords:** Assessment, Outcome measure, Functional measures

## Abstract

**Background:**

Valid and reliable outcome measure enable measurement of health care service impact. There are limited valid and reliable outcome measures for use in podiatry practice to measure the impact of treatment. This research aimed to test the face validity of the AusTOMs for Physiotherapy (AusTOMs-PT), it’s adaptability to podiatry clinical practice and the reliability of its use with podiatrists.

**Methods:**

Stage 1 used a nominal group technique with podiatrists who worked in public and/or private settings. All podiatrists underwent self-directed training in the AusTOMs framework and measures prior to interviews or focus group discussion. Discussion was centred about transferability of the core scales of the AusTOMs-PT and an adjunct measure, AusTOMs for Occupational Therapy (AusTOMs-OT) to podiatry practice.

Stage 2 used 10 case studies representative of people who had foot or ankle concerns. Podiatrists were recruited and trained in the use of the relevant AusTOMs-PT scales. Podiatrists individually scored the cases at two timepoints (1 month apart) using the six scales from the AusTOMs-PT deemed by stage 1 as relevant to podiatry. Intra and inter-rater reliability of scales were determined using intraclass correlation coefficients (ICCs).

**Results:**

Thirteen podiatrists participated in individual or focus group interviews in Stage 1. Consensus was gained on six of the nine core scales adopted from the AusTOMs-PT. These were 1. Balance and Postural Control, 3. Musculoskeletal Movement Related Functions, 4. Neurological Movement Related Functions, 5. Pain, 7. Sensory Functions, 8. Skin Functions. Each core scale rated the functional domains of Impairment, Activity Limitation, Participation Restriction and Wellbeing/Distress relating to that presentation of goals of the person in the case study.

There were 22 podiatrists complete training and scored two rounds of case studies using the six scales in Stage 2. There were 91%(*n* = 20) participants with an intra-rater ICC > 0.5 (moderate or greater). Each domain had an inter-rater reliability of > 0.9 (excellent) during the first round.

**Conclusions:**

The AusTOMs-PT for use in podiatry may be implemented to record change in impairment, function, participation and wellbeing of people receiving podiatry treatment. Podiatry specific training and mentoring, together with repeated use could be expected to improve intra-reliability.

## Introduction

Best practice guidelines promote routine and systematic outcome data collection as part of continually improving care. The measurement of the health outcomes of people who attend health services is imperative in health care and should cyclically feed back into the development of evidence based practice [1]. The importance of collecting this type of data is increasingly apparent as health care funding allocation is tied to person-centered outcome data [2]. Within Australia, there is a recently recognised need and requirement by funders to ensure podiatrists embed validated outcome measures within treatment to record improvement or deterioration subsequent to podiatry care [3, 4]. Funding bodies require these valid outcome measures to highlight the immediate impact or ongoing need for therapy, to ensure the health and functional status of the person receiving care is recorded and that government money is being appropriately utilised.

Much of health practice revolves around the use of diagnostic measures to identify conditions, and guide subsequent treatment paths. Outcome measures are commonly used within public health settings to measure the impact of whole of service care on general health, and used by many other allied health professions to measure their therapy impact. These measures may include, but are not limited to, the Functional Independence Measure [5], the de Morton Mobility Index [6], the Australian Therapy Outcome Measures suite [7] or physical outcome measures such as the 6 min walk test [8] or sit to stand test [9]. These differ greatly from patient report outcome measures (PROMs), such as the Ankle Fracture Outcome of Rehabilitation measure [10]. PROMs commonly are linked to particular conditions, and collected from the patient answering questions about the impact of this condition on their health. There are inherent limitations to both health reported outcome measures being unintendedly manipulated, over or under reported as they are clinician related. Similarly, the limitation of a patient over or under reporting the impact of the condition on their health. There are also differences between both in licencing costs, time taken to administer and participant burden.

Yet there are few outcome measures routinely used in the podiatry profession to measure the impact of care or treatment provided. Studies undertaken relating to foot health or podiatry intervention appear to favour foot and ankle specific tools such as the Foot Health Status Questionnaire [11, 12], Manchester Foot Pain and Disability Index [11] or the Oxford Ankle and Foot Questionnaire [13]. While these tools provide excellent indications of foot health change or quality of life change relating to foot health, they are commonly person rated, condition specific and may not be applicable in settings where a person is unable to rate their own change. These tools are uni- or bi-dimensional, with the outcome of interest being the person’s foot health or quality of life. The majority of foot related tools provide little information for people unable to self-report, for children or where the foot health or goals of treatment do not fit the domains of interest in which the tool was designed.

The Australian Therapy Outcome Measure (AusTOMs) are a suite of measures developed for a number of allied health professions; Occupational Therapy, Speech Pathology and Physiotherapy. They were developed using classical test theory [14] and allow health professionals to score a person’s status across four domains of health and functioning (Impairment, Activity Limitation, Participation Restriction and Distress/Wellbeing). This suite of measures were also developed with a particular focus, and linkage to the International Classification of Functioning, Disability and Health (ICF) [15]. The AusTOMs for occupational therapy (AusTOMs-OT), speech pathology (AusTOM’s - SP) and physiotherapy (AusTOMs - PT) provide a way of scoring change in health and function across all health settings, all conditions and all ages [16]. The scales manual is available for free download from www.austoms.com. Allied health disciplines utilising the appropriate specific profession specific AusTOMS have demonstrated cost-effectiveness during implementation, good psychometric properties, and established psychometrics including minimally clinically importance differences in a variety of conditions [7, 17–19]. Given the deficit of such tools for the podiatry profession, the primary aim of this study was to determine the face validity of the robustly developed AusTOMs for podiatry practice. The secondary aim was to investigate the inter-rater and intra-rater reliability of use of any relevant AusTOMs scales relevant to podiatry practice.

## Methodology

This research used a mixed methods design. Stage 1 used a nominal group technique [20]. Stage 2 tested the inter and intra-rater reliability of the relevant scales and each domain. Ethics approval was provided by Peninsula Health, Monash University, and CQUniversity.

### Participants

Both stages recruited Australian podiatrists working in both public and private settings. Inclusion criteria for participation in either stage was current registration with the Australian Health Practitioner Regulation Agency, and a direct service delivery role in any clinical setting for a minimum of 8 h per week. All podiatrists provided written participation consent to undertake training, and have their voice recorded during subsequent focus group or interview. A separate recruitment and consenting process was undertaking for Stage 2, with the same eligibility criteria.

### Instruments

#### Stage 1

During this stage, podiatrists undertook self-directed training provided with a video link and user guide from the AusTOMs website [21]. Four case studies (Supplementary File 1) were developed by the research team. These four case studies were decided by the research team cover a breadth of common presentations to any podiatry clinician in either the public or private setting. These cases were trialled during development with three podiatrists not involved with the study for feedback on their accuracy of podiatry presentations. During their development, the cases were scored using four difference scales of the AusTOMs-PT. Once participating podiatrists completed the training, they were provided with a hardcopy of the AusTOMs-PT user guide, and the 9 AusTOMs-PT and the 12 AusTOMs-OT scale cards [21].

Each scale card requires the user to score a presentation on four domains: Impairment, Activity limitation, Participation restriction and Distress/wellbeing. Scores are on a eleven-point Likert scale ranging from 0 being unable or the worst, through to 5 being no impact with half points accepted. Both the AusTOMs-PT and AusTOMs-OT have established validity and validity [7, 18, 22, 23] with both tools established reliable when used by novice or experienced health professionals.

#### Stage 2

This stage utilised the scales agreed upon within Stage 1. An additional six case studies were developed presenting clinical information based on a wide range of commonly presenting conditions to podiatry services in different settings to add to the four cases developed during face validity testing. The cases were again developed by three podiatrists with public and private clinical experience and deemed representative of the types of cases seen in either setting based on recent presentations in either setting. All research team members provided feedback and approved the case studies for use in *Stage 2*. Cases were again piloted with an additional two podiatrists external to the study to ensure ease of reading and clarity in the information, with only minor language changes made as required. Table 1 gives a brief overview of the cases.

**Table 1:** Description of case studies associated the scale scored, for the AusTOMs - PT for use in Podiatry

Case	Description	Scale scored
1	Cory, Age 10 Heel pain with associated reduction in participation	4. Pain
2	Lisa, Age 40 Progressive neurological condition causing foot deformity	3. Neurological Movement Related Functions
3	Trent, Age 58 Type 2 Diabetes with suspected Charcot Neuroarthropathy	5. Sensory Functions
4.	Edith, Age 90 Pressure injury at heel	6. Skin Function
5.	Samantha, Age 11 Idiopathic toe walking	1. Balance and postural control
6.	Gary, Age 80 General foot care needed due to inability to reach feet	2. Musculoskeletal Movement Related Functions
7.	Sherrie, Age 54 Peripheral neuropathy	5. Sensory Function
8.	Richard, Age 62 Recent amputation of second toe on left foot	6. Skin Function
9.	Sue, Age 56 Right acute plantar heel pain	4. Pain
10	Jim, Age 68 Recent fall and associated hip fracture	1. Balance and postural control

### Procedures

#### Stage 1

Podiatrists were recruited from direct email to a public health-care service podiatry department in Victoria, Australia, in addition to social media advertising. This public health service provides care in acute, subacute and community settings and to all ages and conditions. No data were collected on the types of patients seen in private practice settings. Podiatrists were screened to ensure they met the inclusion criteria and included as a first come, first enrolled in either a focus group or individual interview depending on their personal preference. Podiatrists were introduced to the required training prior to interview [21] and asked to provide basic demographic data on their recency of practice and primary practice setting.

Stage 1 involved a nominal group technique to gain feedback on the outcome measure’s applicability. This technique is a highly structured method of focus group using structured discussion and techniques to ensure all participants are equally involved. Individual interviews used the same questioning sequence. The group facilitator/interviewer conducted all focus groups, interviews and had additional training and qualifications in mediation and facilitation. This technique used four key stages: silent generation, round robin, clarification and voting in the focus group [24]. In the individual interviews, the same process was adopted and where appropriate the results and comments from the focus group was read out to ensure the podiatrist was provided with the same additional dialogue. All podiatrists had access to the written outcome measurement tools and the scored podiatry cases.

Podiatrists were firstly asked: “*Using the AusTOMs for Physiotherapy headings, are these accurate reflections of therapy areas covered podiatry?”* Podiatrist responses were recorded. Podiatrists were then provided with the case studies and asked: “*On reading the cases, which of the AusTOMs for Physiotherapy domains are applicable to these common podiatry presentations?”* and *“On reading the cases – is there a Scale missing that is podiatry profession specific, and must be included?”* Podiatrists responses were audio-recorded.

#### Stage 2

Stage 2 was conducted 8 months after Stage 2 to allow development of materials. Podiatrists initially underwent training in the use of AusTOMs if they had not previously been trained while participating in Stage 1 [21] or were urged to watch the training video they were unable to remember how to score or require more support than just the training manual. Participants provided basic demographic data including their gender, recency of practice and primary practice setting. Podiatrists were electronically provided with the 10 podiatry specific cases studies and asked to electronically score these in Qualtrics [25]. Completion was electronically monitored by the research team. Podiatrists were asked to destroy any notes they made during the process of scoring and confirmed this with the research team at their completion of round 1. All podiatrists were then contacted 4 weeks after their first completion of the ten cases and provided with a new Qualtrics link to undertake the same process. The same cases were again electronically presented in a random order. Podiatrists again scored the cases on each domain of Impairment, Activity Limitation, participation Restriction and Distress/Wellbeing. A nominal $20AUD gift card was provided as an appreciation for their time.

### Data analysis

All podiatrist participant data for Stages 1 and 2 were described in frequencies (%), means (SD), ranges and median (IQR). In Stage 1, participant recordings were transcribed verbatim. Consensus was determined as a minimum of 70% of podiatrists providing the same response of scale applicability at the end of the combined focus group and interviews in the voting process.

During the reliability analysis, Likert scale data from each domain were treated as continuous data. Previous reliability studies of the AusTOMs have utilised this approach [19, 22, 23]. The intra-rater reliability for each domain at the two timepoints was determined with the intraclass correlation coefficient (ICC) [Model 2,1] with a 95% confidence interval (95% CI). Inter-rater reliability for each domain was determined at the first timepoint only using an ICC (Model 2, k) and 95% CI. The minimum sample size of 18 was determined to provide 80% power of detecting a ICC of 0.9 with a two-tailed alpha = 0.05 for the inter-rater reliability analysis [26]. To interpret the ICC data, ICC ranges < 0.5 = poor reliability, 0.5 to 0.75 = moderate reliability, 0.76 to 0.9 = good reliability, and > 0.90 = excellent reliability [26]. All data were analysed with Stata 15 [20].

## Results

In Stage 1, there were 13 participants provide feedback on the validity of the AusTOM-PT and the scale suitability for common podiatry clinical presentations. A single focus group was run with 10 participants. The three participants who could not attend had individual interviews within 2 weeks of the focus group. The majority of participants were females (*n* = 10, 77%) in Stage 1, and participants recency of practice ranged between 1 and 34 years with a median (IQR) of 8 (4, 14.75) years. Participants had diverse clinical experience, with 3 (24%) working only in private practice settings, 5 (38%) working only in public health settings acute, subacute or community based) and the remaining 5 (38%) podiatrists working in both public and private settings. Podiatrists were in consensus (*n* = 13, 100%) that the scale headings from the AusTOM’s-PT provided an accurate reflection of treatment areas for podiatry presentations. Participants were in also in consensus (n = 13, 100%) that six AusTOM’s-PT scales were appropriate in the podiatry settings, these being:

1. Balance and Postural Control,

3. Musculoskeletal Movement Related Functions

4. Neurological Movement Related Functions

5. Pain

7. Sensory Functions

8. Skin Functions

There were two participants (15%) who indicated that the AusTOMs-OT scale *7. Self Care* may apply to podiatry, but that this scale had similarities in its measurement and descriptions to the AusTOMs – PT Scale *8. Skin Functions* or *5. Pain*. The AusTOMs-OT scale 7 was not included in the final outcome measurement tool that underwent reliability testing in Stage 2.

There were 22 podiatrists recruited for Stage 2 reliability testing with majority being female, (*n* = 14, 63%), and a mean age of 32.8 (8.2) years. Seven of these podiatrists participated in Stage 1. The majority of podiatrists were from Victoria (n = 14, 63%), and two podiatrists (9% each) from each state of Queensland, New South Wales and Tasmania, and one (5%) from Western Australia and one (5%) from South Australia. All podiatrists scored the cases at the two timepoints between 4 and 6 weeks apart. Participants had diverse clinical experience, with 10 (45%) working only in private practice settings, 7 (32%) working only in public health settings (acute, subacute or community based) and the remaining 5 (23%) podiatrists working in both public and private settings. Participants recency of practice ranged between 1 and 19 years with a median (IQR) of 10 (5, 13) years of clinical experience. Table 2 provides individual reliability scores. There were 9 (41%) podiatrists who had a good or excellent reliability for the Impairment domain, and an additional 9 (41%) podiatrists who had moderate reliability for the Impairment domain (Table 2). There were 18 (82%) of podiatrists who had a good or excellent reliability for the Activity Limitation domain (Table 2). There were also 14 (64%) who had a good or excellent reliability for the Participation Restriction domain and 21 (95%) who had a good or excellent reliability for the Distress/Wellbeing domain.

**Table 2:** Intra-rater reliability intraclass cirrelation coefficient [95% confidence interval] for all scales and all domains

Participant	Domain			
	Impairment	Activity Limitation	Participation Restriction	Distress/Wellbeing
1	0.49 [− 0.13, 0.83]	0.77 [0.35, 0.93]	0.91 [0.69, 0.97]	0.89 [0.65, 0.97]
2	0.71 [0.23, 0.95]	0.49[−0.18, 0.84]	0.66 [0.14, 0.90]	0.73 [0.26, 0.92]
3	0.61 [0.05, 0.88]	0.88 [0.61, 0.97]	0.65 [0.12, 0.99]	0.93 [0.75, 0.98]
4	0.83 [0.50, 0.95]	0.85 [0.55, 0.96]	0.84 [0.51, 0.95]	0.89 [0.64, 0.97]
5	0.80 [0.41, 0.94]	0.67 [0.14, 0.90]	0.64 [0.10, 0.89]	0.87 [0.60, 0.96]
6	0.53 [−0.07, 0.85]	0.94 [0.80, 0.98]	0.46 [−0.15, 0.83]	0.80 [0.42,0.94]
7	0.80 [0.43, 0.94]	0.93 [0.78, 0.98]	0.89 [0.64, 0.97]	0.78 [0.37, 0.94]
8	0.89 [0.65, 0.97]	0.83 [0.49, 0.95]	0.82 [0.45, 0.95]	0.76 [0.33, 0.93]
9	0.69 [0.18, 0.91]	0.92 [0.75, 0.98]	0.81 [0.44, 0.94]	0.90 [0.66, 0.97]
10	0.66 [0.13, 0.90]	0.85 [0.52, 0.95]	0.76 [0.33, 0.93]	0.88 [0.61, 0.96]
11	0.63 [0.09, 0.89]	0.49 [−0.12, 0.84]	0.41 [−0.21, 0.81]	0.83 [0.49, 0.95]
12	0.77 [0.35, 0.93]	0.92 [0.74, 0.98]	0.89 [0.66, 0.97]	0.39 [−0.24, 0.80]
13	0.80 [0.41, 0.94]	0.82 [0.48, 0.95]	0.90 [0.69, 0.97]	0.89 [0.66, 0.97]
14	0.81 [0.45, 0.95]	0.93 [0.75, 0.98]	0.87 [0.61, 0.97]	0.92 [0.73, 0.98]
15	0.77 [0.36, 0.94]	0.93 [0.76, 0.98]	0.77 [0.35, 0.93]	0.97 [0.91, 0.99]
16	0.69 [0.18, 0.91]	0.87 [0.60, 0.96]	0.95 [0.85, 0.98]	0.94 [0.79, 0.98]
17	0.71 [0.22, 0.91]	0.76 [0.32, 0.93]	0.65 [0.11, 0.89]	0.92 [0.75, 0.98]
18	0.39 [−0.24, 0.80]	0.83 [0.49, 0.95]	0.62 [0.06, 0.88]	0.78 [0.36, 0.94]
19	0.33 [−0.31, 0.77]	0.91 [0.69, 0.97]	0.78 [0.38, 0.94]	0.80 [0.41, 0.94]
20	0.51 [−0.09, 0.84]	0.69 [0.17, 0.91]	0.65 [0.11, 0.89]	0.87 [0.59, 0.96]
21	0.90 [0.68. 0.97]	0.92 [0.73, 0.97]	0.79 [0.40, 0.94]	0.97 [0.91, 0.99]
22	0.37 [−0.27,0.79]	0.92 [0.72, 0.97]	0.83 [0.49, 0.95]	0.80 [0.41, 0.94]

The inter-rater reliability was calculated at the first timepoint, with all domains demonstrating excellent inter-rater reliability (Impairment: ICC = 0.98, 95% CI = 0.95 to 0.99, Activity Limitation: ICC = 0.98, 95%CI = 0.96 to 0.99, Participation Restriction: ICC = 0.96, 95%CI = 0.92 to 0.99), Distress/Wellbeing; ICC = 0.99, 95%CI 0.97 to 0.99).

## Discussion

Podiatrists are increasingly focusing their clinical care on service provision to enhance participation and to maximise the wellbeing of individuals seeking care. This is a shift from a disease-based model of care to one of a participatory or wellness model for the profession. Consequently, it is increasingly important for outcome measurements in the clinical setting to capture change. The AusTOMs-PT for use in Podiatry provides six scale across four domains. Each scale and domain provide insight into the improvement or deterioration of a person who is receiving podiatry care. The preliminary validity and reliability findings indicate that these scales are suitable to commence trialling as outcome measures for any type of podiatry service across Australia and potentially in similar international health settings.

The results achieved in this study demonstrate relatively high levels of reliability among this group of podiatrists. These findings were similar or higher than the recent AusTOMs-OT reliability study [18]. The AusTOM’s-OT study adopted a similar case study approach with 31 occupational therapists across Australia and the UK. Past research has found a small number of differences seen between professions in rating different domains and their reliability using the AusTOMs in this manner. Physiotherapists have been noted to have some difficulty rating ‘ Participation Restriction’ and ‘Distress/well-being’ based on paper case studies [27]. Occupational therapists have demonstrated difficulty in rating the Impairment domain [18]. While there was excellent reliability between podiatrists in the first round, the intra-rater ICC scores for the Impairment domain were often lower than the other domains for many individual participants. This may have been due to the complexities not being fully explained in a theoretical and a paper-based case study. These case studies miss the richness of data that having a person recall their health problem and its impact, brings to a consultation.

We acknowledge that the using paper-based case studies was the primary limitation of this study. In addition, the training provided to podiatrists used theoretical case studies relevant to Occupational Therapists. This may have impacted how the podiatrists understood the use of the tool and may have affected the reliability. Development of podiatry specific training, with podiatry specific case examples for tool use may ameliorate this. This pragmatic approach during the study was based on the complexity of undertaking a history from a person seeking podiatry care from multiple podiatrists and at multiple timepoints. Videos have previously been suggested as a mechanism to improve reliability of the case study presentation, however this approach was not possible for this unfunded study. An additional limitation was that scales only provide a snapshot of the person’s presentation and not a linear scale of change of this presentation over time based on any treatment. The advantage of this is that is only took a moment for the podiatrists in the study to use the tool however, this mock scenario use would need adapting for clinical practice.

A strength of this study was the varied cases presented to podiatrists. We believe the variability of these cases are an accurate representation of people who seek podiatry practice treatment across the many sectors. The author group has international representation and this enabled the case development to be potentially appropriate for podiatrists who work in countries other than Australia. Future research may consider specific conditions to determine minimally important clinical differences to better understand therapy impact.

Given the promising reliability of the AusTOMs – PT for use in Podiatry, there is scope for this outcome measure to be embedded in clinical practice and future research. This may include face validity in other countries, development of minimally important clinical differences for specific conditions and large datasets to understand the overall impact of podiatry-based services in difference care settings.

## Conclusion

The AusTOMs-PT for use in Podiatry is a valid and reliable outcome measure. It may be implemented in private or public podiatry services to record change in impairment, function, participation and wellbeing of people receiving podiatry treatment. This tool may be of assistance to podiatrists who are required to use outcome measures for funding requirements within the Australian health care setting such as the disability (e.g. National Disability Insurance Scheme) and health care sectors (e.g. Department of Veteran’s Affairs). Specific training and repeated use may be expected to improve the intra-reliability in the domain of impairment. Further research could should consider the use of this outcome measure as part of the suite of measures to understand the impact of podiatry treatment.

## Supplementary information

**Additional file 1.** Example Case Studies For Podiatry.

## Data Availability

Raw data is available from authors on request.

## Abbreviations

ICC: Intraclass coefficient
SEM: Standard error of measurement
AusTOMs: Australian Therapy Outcome Measures
AusTOMs-PT: Australian Therapy Outcome Measures - Physiotherapy
AusTOMs-OT: Australian Therapy Outcome Measures – Occupational Therapy
