# Supplementary material for: Validity and reliability of the Australian Therapy Outcome Measures – Physiotherapy, for podiatry (AusTOMs-PT for use in podiatry)
Source: J Foot Ankle Res. 2020 Apr 25;13:17. doi: 10.1186/s13047-020-00385-0 (PMC7183624; doi:10.1186/s13047-020-00385-0)
Supplement: Supplementary file 1 — Additional file 1. Example Case Studies For Podiatry. [file 13047_2020_385_MOESM1_ESM.docx]

**Example Case Studies For Podiatry (V1, 27/07/2018)**

**Case Study 1:**

**AusTOMs for Physiotherapy**

**Scale 5 - Pain**

**Background**

Cory is a 10-year-old, typically developing boy. He started with pain, relatively quickly, 6 weeks ago after a recent growth spurt. Prior to this pain, he was fully active and was enjoying his sports. The pain is now affecting his ability to fully participate in his sport. He lives at home with his parents and his older brother. He attends school and has full attendance. Cory plays basketball twice a week and enjoys school sporting activities during the week. The podiatrist is seeing Cory to investigate why he is getting the pain and how this can be managed so he can return to full activity levels.

**Impairment**

Cory presents with pain in his left heel. The area is not hot, swollen or red. There is normal range of movement in the foot and limitation of range into ankle dorsiflexion on the left side, but only because he stops it moving due to it hurting during testing. Neurological testing is in normal limits. In gait, the pain is causing him to limit his heel contact. On asking him to contact with his heel, this causes pain. The pain increases after sporting activity, this causes him to limp more than he does when walking. His pain is better controlled when he wears trainers and or doesn't exercise for longer than 20 minutes.

**Activity**

Cory attempts all his usual activities. He finds that prolonged activity involving running and jumping starts to cause him pain. This affects his speed and agility in performing these tasks required for his sport. He is self selecting out of sport and reducing the duration of his activities. At the end of a game he struggles to walk comfortably and needs more time to complete this activity.

**Participation**

Cory has not had any time away from school and has attended all his scheduled activities; however, last week he was unable to compete in his school cross-country race. Sometimes Cory has had to finish basketball training early due to the pain in his heel, without the practice he worries he will be held back. Cory finds that he is unable to get through a whole 40 minute game of basketball. Cory feels he is not keeping up with his peers as they can do things better than he can. He is frustrated that he can't spend as long with his friends. His family are able to drive him home from activities which help him continue to participate.

**Wellbeing**

Cory is determined to carry on participating but when the pain gets too bad, this prevents him. He gets upset sometimes when his foot is hurting and is beginning to worry it will never get better. He is relieved that wearing trainers helps a little. His parents have assured him that someone will be able to help and have sought professional help. Cory is fearful that he may miss out on his basketball tournament. He is worried his team may choose other people to play.

**Write your scores in the Table below**

**Scale 5: Pain**

| **Domain** | **Score** |
| --- | --- |
| Impairment | 4 |
| Activity Limitation | 4 |
| Participation Restriction | 4 |
| Distress/ Wellbeing | 4 |

**Case Study 2**

**AusTOMs for Physiotherapy
Scale 4: Neurological Movement Related**

**Background**

Lisa is a 40 year old lady who has a genetic neurological condition which has caused progressive physical changes over time. Lisa lives at home with her husband and her 14 year old son. She works part-time, 2 days a week as a receptionist. She has a close network of family and friends. Lisa has a severe pes cavus foot structure and requires ongoing podiatry input.

**Impairment**

Due to her neurological condition, Lisa has experienced progressive muscle wasting with reduced muscle strength in both of her distal legs and feet. She is unable to walk on her heels and on walking tip toe, she is unstable. The loss of strength affects her active range of movement and the ankle and mobility in her lower limbs. She has reduced knee and ankle reflexes and sensory impairment to her midfoot. In gait, she has an atypical walking pattern which also demonstrates instability. She has a bilateral rigid, cavus foot deformity associated with weakness. The foot is unable to adapt to sudden variations in movement easily.

**Activity Limitations**

Lisa is independent with ambulation. She has difficulty with stairs and uneven surfaces. To aid ambulation, she always wears custom footwear and supportive orthotic devices which help her foot comfort, confidence, stability and safety. Lisa also manages difficult activities by using handrails and walking mindfully and slower. She is able to complete most activities of daily living (ADL’s) but requires some assistance from her family members if the environment and the task are not ideal e.g. walking on uneven surfaces in low light or carrying heavy objects.

**Participation**

Lisa is able to continue working part time and her neurological condition doesn't require her to take time off work. She is financially stable. Lisa has adjusted her leisure activities to fit her abilities and has found a balance which feels right for her. She has her own car and has the confidence to use all forms of public transport independently. She is able to maintain a healthy social life with friends and family and doesn't feel restricted by her circumstances.

**Wellbeing**

Lisa has a good understanding of her neurological condition. She values the network of family and friends. Overall, she is a happy and cheerful lady who is able to cope with most situations. She understands her own limitations.

**Write your scores in the Table below.**

**Scale 4: Neurological Movement Related**

| **Domain** | **Score** |
| --- | --- |
| Impairment | 2 |
| Activity Limitation | 2.5 |
| Participation Restriction | 4 |
| Distress/ Wellbeing | 5 |

**Case Study 3
AusTOMs for Physiotherapy
Scale 7: Sensory Functions**
**Background**

Trent is a 58-year-old male he has had Type 2 Diabetes for over 15 years. He has poor glycaemic control and a BMI of 37. He reports his left foot as being red, hot and swollen for about 5 days.  He has taken time off work due to a change in his foot condition. He presents to podiatry with suspected active Charcot neuroarthropathy on his left foot. He lives at home with his wife and two teenage children and he has an active, full time job in the trade industry.

**Impairment**

Trent's vascular assessment doesn't reveal concern of calcification. His skin is intact and his left foot is red, swollen and hot with over 2 degrees of difference in temperature. He has a complete loss of protective sensation in both of his feet and sensory loss extends to the distal 1/3 of the calf on 10g monofilament testing. Vibration sensation is reduced in both feet. There is asymmetrical foot posture with evidence of midfoot deformity on the left foot.

**Activity Limitations**

Trent’s drives a manual car, he acknowledges his driving ability is compromised due to him not being able to feel the pedals. This has been gradually changing over time. He is finding it difficult to ambulate throughout his house as it is multi-level and has one flight of internal stairs. He will be required to reduce his weight-bearing status during treatment which will impact his ADL’s and will most likely require supportive devices and some assistance from his family.

**Participation**

Trent is currently taking time off work so is less active on his feet. He is unsure how much time off is required, this is causing him worry as his pay is only protected for one month and he has no insurance to protect him after this time. He has modest savings. His wife works part-time. He is usually quite active with his children on the weekends and is finding it difficult to accept that commitments may change.

**Wellbeing**

He is fearful that he may not be able to provide an income for his family if there is substantial time away from work and that he may lose employement. Both his wife and children are solely dependent on his income.

**Write your scores in the Table below.**

**Scale 4: Neurological Movement Related**

| **Domain** | **Score** |
| --- | --- |
| Impairment | 2 |
| Activity Limitation | 3 |
| Participation Restriction | 3.5 |
| Distress/ Wellbeing | 4 |

**Case Study 4**

**AusTOMs for Physiotherapy
Scale 8: Skin Functions**
**Background**

Edith is a 90 year old lady. She is widowed and lives at home by herself. Whilst in hospital care she sustained a pressure injury on her heel, podiatry have been providing wound and pressure care whilst she is in hospital. Edith is finding it difficult to complete her rehabilitation goals due to the current pressure injury. She is due for discharge in one week.

**Impairment**

Edith has limited mobility and spends over 23 hours a day in a bed or chair, therefore she is at high risk of further ulceration. She demonstrates muscle weakness sitting to standing. She has a grade 3 wound on her left heel which is not infected, it has moderate exudate and showing signs of slow but progressive improvement. She generally has very poor skin integrity with reduced perfusion to her lower limbs, therefore she is likely to have delayed healing. She is experiencing moderate amounts of pain, swelling and inflammation at the pressure injury site. Consequently, on standing, her pain increases and instability occurs.

**Activity Limitations**

Edith finds her rehabilitation difficult to complete now with her recent reduction in mobility, strength and current pressure injury. She needs assistance sitting to stand with zimmer-frame and requires a high backed chair with arms. She is having trouble walking up to 10 meters on flat surfaces. Transferring and showering requires assistance from nursing. Living alone, she worries that the hospital in the hospital in the home care arranged will fall short of her frequent needs that have been met in hospital.

**Participation**

Edith enjoys watching television, listening to the radio or reading in her chair during the day. She keeps in touch with her friends and family by telephone. Some are able to visit each week. She is keen to get back in her routine and would like to regain the independence of personal care. Edith will need ongoing care once discharged home with regular nursing and home help visits to help with showering and wound care. She will also require appliances and adaptations to provide pressure care at home.

**Wellbeing**

Edith is anxious that the pressure ulcer this will affect her planned discharge date, she is keen to return home rather than an aged care facility. Her confidence has been lowered as the event has left her with less autonomy and privacy. She is unsure how she will physically cope at home with less frequent support and emotionally with less independence.

**Write your scores in the Table below.**

**Scale 8: Skin Functions**

| **Domain** | **Score** |
| --- | --- |
| Impairment | 2 |
| Activity Limitation | 3 |
| Participation Restriction | 3.5 |
| Distress/ Wellbeing | 3 |
